# The Role of BACH2 in T Cells in Experimental Malaria Caused by *Plasmodium chabaudi chabaudi* AS

**DOI:** 10.3389/fimmu.2018.02578

**Published:** 2018-11-06

**Authors:** Chelsea L. Edwards, Marcela Montes de Oca, Fabian de Labastida Rivera, Rajiv Kumar, Susanna S. Ng, Yulin Wang, Fiona H. Amante, Kohei Kometani, Tomohiro Kurosaki, Tom Sidwell, Axel Kallies, Christian R. Engwerda

**Affiliations:** ^1^QIMR Berghofer Medical Research Institute, Brisbane, QLD, Australia; ^2^School of Medicine, University of Queensland, Brisbane, QLD, Australia; ^3^Department of Biochemistry, Banaras Hindu University, Varanasi, India; ^4^School of Natural Sciences, Griffith University, Nathan, QLD, Australia; ^5^Laboratory for Lymphocyte Differentiation, RIKEN Center for Integrative Medical Sciences (IMS), Kanagawa, Japan; ^6^Laboratory of Lymphocyte Differentiation, WPI Immunology Frontier Research Center, Osaka University, Osaka, Japan; ^7^Department of Microbiology and Immunology, The Peter Doherty Institute of Infection and Immunity, University of Melbourne, Melbourne, VIC, Australia; ^8^The Walter and Eliza Hall Institute of Medical Research, Melbourne, VIC, Australia

**Keywords:** BACH2, malaria, protozoan, T cells, inflammation

## Abstract

BTB and CNC Homology 1, Basic Leucine Zipper Transcription Factor 2 (BACH2) is a transcription factor best known for its role in B cell development. More recently, it has been associated with T cell functions in inflammatory diseases, and has been proposed as a master transcriptional regulator within the T cell compartment. In this study, we employed T cell-specific *Bach2*-deficient (B6.*Bach2*^Δ*T*^) mice to examine the role of this transcription factor in CD4^+^ T cell functions *in vitro* and in mice infected with *Plasmodium chabaudi* AS. We found that under CD4^+^ T cell polarizing conditions *in vitro*, Th2, and Th17 helper cell subsets were more active in the absence of *Bach2* expression. In mice infected with *P. chabaudi* AS, although the absence of *Bach2* expression by T cells had no effect on blood parasitemia or disease pathology, we found reduced expansion of CD4^+^ T cells in B6.*Bach2*^Δ*T*^ mice, compared with littermate controls. Despite this reduction, we observed increased frequencies of Tbet^+^ IFNγ^+^ CD4^+^ (Th1) cells and IL-10-producing Th1 (Tr1) cells in mice lacking *Bach2* expression by T cells. Studies in mixed bone marrow chimeric mice revealed T cell intrinsic effects of BACH2 on hematopoietic cell development, and in particular, the generation of CD4^+^ and CD8^+^ T cell subsets. Furthermore, T cell intrinsic BACH2 was needed for efficient expansion of CD4^+^ T cells during experimental malaria in this immunological setting. We also examined the response of B6.*Bach2*^Δ*T*^ mice to a second protozoan parasitic challenge with *Leishmania donovani* and found similar effects on disease outcome and T cell responses. Together, our findings provide new insights into the role of BACH2 in CD4^+^ T cell activation during experimental malaria, and highlight an important role for this transcription factor in the development and expansion of T cells under homeostatic conditions, as well as establishing the composition of the effector CD4^+^ T cell compartment during infection.

## Introduction

BTB and CNC Homology 1, Basic Leucine Zipper Transcription Factor 2 (BACH2) is a leucine zipper transcription factor known for its role in B cell development. It is also involved in antibody class switch recombination and somatic hyper-mutation (1). More recently, it has been associated with T cell function in a number of inflammatory diseases, and as such, has been described as a master transcriptional regulator within the T cell compartment (2, 3). *BACH2* dysregulation has been associated with a number of immune disorders, including tumor suppression and control of B cell lymphomas (4). However, in some cancers it was mutated or fused with other genes leading to dysregulated expression of *BACH2* itself or BACH2 fusion protein (5, 6).

*BACH2* is often down-regulated in inflammatory disorders. For example, CD4^+^ T cells from coeliac disease patients had down-regulated *BACH2* expression associated with inflammation (7). Interestingly, *IFN*γ was highly expressed in these CD4^+^ T cells, suggesting that BACH2 may play a role in regulating *IFN*γ expression. Several genome wide association studies have also found significant associations between the presence of single nucleotide polymorphisms in *BACH2* and susceptibility to inflammatory diseases, including rheumatoid arthritis, Crohn's disease, asthma, and multiple sclerosis (8–11). In a mouse model of multiple sclerosis (experimental autoimmune encephalomyelitis; EAE), *Bach2* was down-regulated in Th17 cells and expression was negatively associated with disease severity (12). Another study showed that *Bach2* was significantly down regulated in T cells during EAE, and this correlated with increased *Foxp3* methylation and reduced *Foxp3* expression, suggesting BACH2 influences epigenetic modification of the *Foxp3* promoter region to support thymic-derived FoxP3^+^ regulatory T (Treg) cell development and expansion (13).

Other studies have identified additional roles for BACH2 in regulating T cell homeostasis (2, 14, 15). Control of T cell numbers is critical for immune homeostasis, and dysregulation can result in immune disorders (16–18). As mentioned above, *Bach2* expression was essential for the stability and function of Treg cells, but also plays a role in the differentiation of CD4^+^ T cells into effector lineages, such as Th1, Th2, and Th17 cells (2, 14, 15). For example, *Bach2* knockout mice developed a Th2 cell-dependent lung disease, associated with enhanced Th2 cell cytokine production and lung inflammation (15), indicating a requirement for BACH2 in controlling Th2 cell differentiation and/or tissue recruitment. BACH2 has also been shown to promote Th1 cell responses over Th2 cell responses during infection. In a mouse model of *Listeria monocytogenes* infection, loss of BACH2 enhanced Th2 cell responses while reducing Th1 cell development (14). *Prdm1* (encoding BLIMP1) expression was increased in T cells from *Bach2* knockout mice, suggesting BACH2 may suppress T cell *Prdm1* expression (14). Thus, a potential mechanism by which BACH2 impacts CD4^+^ T cell differentiation is by suppressing *Prdm1* expression. This would normally promote Th2 cell differentiation by down-regulating Th1 and T follicular helper (Tfh) cell lineage genes, such as *Tbx21* and *Bcl6*, respectively (19). However, BACH2 also appeared to promote CD4^+^ T cell regulatory phenotypes over other CD4^+^ T cell subsets. This was supported by the unrestrained Th2 cell-mediated wasting disease observed in *Bach2* knockout mice, along with upregulation of Th1, Th2, and Th17 cell-associated genes, when CD4^+^ T cells from these mice were polarized under relevant conditions (2). BACH2 can also suppress CD8^+^ T cell function, although this was shown to be indirect, and occurred via the inhibitory actions of Treg cells (20). Thus, in autoimmune disease and *in vitro* cell culture assays, BACH2 promotes development of a regulatory CD4^+^ T cell phenotype, while suppressing development of effector CD4^+^ T cells through both cell intrinsic and extrinsic mechanisms. Whether this also occurs in parasitic diseases is unknown.

Intracellular protozoan parasites that cause diseases such as malaria and leishmaniasis generally require a pro-inflammatory immune response mediated by Th1 cells for control of parasite growth (21). In the case of *Plasmodium* species that cause malaria, a robust T follicular helper (Tfh) cell response is also needed to generate protective anti-parasitic antibodies (22–25). However, disease often develops because these responses are either impaired or dysregulated. Recently, Foxp3^−^ IL-10-producing Th1 cells (type 1 regulatory; Tr1), rather than thymus-derived FoxP3^+^ CD4^+^ regulatory T (Treg) cells, have also been recognized to play important roles in determining the outcome of protozoan parasitic diseases, including malaria, leishmaniasis and toxoplasmosis (26–29). IL-10 production by Tr1 cells has been shown to be governed by BLIMP (30, 31), and we recently showed that *Prdm1* expression by T cells enhanced Tr1 cell development, while suppressing Th1 cell expansion (28). This was associated with enhanced parasite burden and increased morbidity in mouse models of malaria and visceral leishmaniasis (VL) (28). Because BACH2 is thought to suppress *Prdm1* expression (32), we hypothesized that BACH2 would antagonize BLIMP1 activity in these diseases, resulting in opposing outcomes to *Prdm1*-deficient mice. BACH2 has predominantly been investigated using *Bach2*-deficient mice, which have not allowed the importance of cell, tissue or temporal expression of BACH2 to be examined. Therefore, we employed T cell-specific *Bach2* knockout mice to investigate the role of BACH2 in protozoan parasitic infections.

## Materials and methods

### Infections and quantification of parasite burden

One passage mouse was infected with 200 μL cryo-preserved *P. chabaudi chabaudi* AS parasitised red blood cell (pRBC) inoculum via intravenous tail injection. When passage parasitemia reached 2–4% (typically 2–4 days post inoculation), blood was harvested and prepared for inoculation of experimental mice. Briefly, passage mice were euthanized using CO_2_ inhalation, blood was harvested via cardiac bleed and washed in media {5IU heparin (Pfizer, NSW, Australia), 1% (w/v) penicillin/streptomycin [Gibco (Thermo Fischer, Walther, MA, USA)], in RPMI}. The concentration of pRBC was adjusted to 5 × 10^5^ per mL in RPMI/PS. Experimental mice were infected with 1 × 10^5^ iRBC via intravenous (i.v.) tail injection.

Parasitemia was monitored via flow cytometry. Briefly, one drop of blood was collected into 200 μL of media. Fifty microliter of diluted blood was incubated with 50 μL of Syto84 [5 μM, Life Technologies (Thermo Fischer)] and Hoechst33342 [10 ug/mL, Sigma (St Louis, MO, USA)] for 30 min, at room temperature, protected from light. This was then diluted out to 6 times the original volume with RPMI, and acquired on one of three BD flow cytometers (Canto II, Fortessa 4, or Fortessa 5). FlowJo software (v.8, Treestar, CA, USA) was used to quantitate parasitemias.

*L. donovani* (LV9; MHOM/ET/67/HU3) was maintained by passage in B6.*Rag1*^−/−^ mice. Amastigotes were isolated from chronically infected passage animals. Experimental mice were infected by injection of 2 × 10^7^ amastigotes i.v., via the lateral tail vein. Mice were culled at different time post-infection (p.i.) indicated in the text by CO_2_ asphyxiation and bled via cardiac puncture. Spleens were removed and livers perfused then removed, with parasite burden determined by qPCR, as previously described (33, 34). Hepatic and splenic mononuclear populations were isolated as previously described (28, 35).

### Mice

Inbred female C57BL/6 and congenic B6.CD45.1 mice, 6 weeks of age, were purchased from the Animal Resource Center (ARC; Canning Vale, WA, Australia). B6.*Cd4*-*Cre* transgenic mice (36) were crossed with B6.*Bach2*-floxed transgenic mice (37) to generate T cell-specific BACH2-deficient C57BL/6 (B6.*Bach2*^Δ*T*^) mice. Littermates lacking the *Cd4-cre* transgene (B6.*Bach2*^*fl*/*fl*^) were used as controls. It should be noted that the *Bach2* gene will be depleted in both CD4^+^ and CD8^+^ T cells in these animal due to expression of both CD4 and CD8 on double positive thymocytes during T cell development. Female mice were used in all experiments and were age-matched, and bred and maintained in-house at QIMR Berghofer (Brisbane, Australia) under pathogen-free conditions. All animal procedures were conducted with the approval of the QIMR Animal Ethics Committee under the animal ethics number A02-634M and in accordance with the “Australian Code of Practice for the Care and Use of Animals for Scientific Purposes” (Australian NHMRC, Canberra).

### *In vitro* stimulation of CD4^+^ T cells

CD4^+^ T cells were isolated from spleens using the Miltenyi mouse CD4^+^ T cell isolation kit, according to manufacturer's guidelines (Miltenyi, Biotec, Bergisch Gladbach, Germany). CD4^+^ T cells (4 × 10^5^/well) were then cultured with αCD28 (1 μg/mL, clone 37.51, BioLegend, San Diego, CA) and plate bound αCD3ε mAb (wells coated with 1 μg/mL for 4 h at 37°C, 5% CO_2_, clone 145-2C11, BioLegend) in a 96-well plate, for 5 days (3 days for Tr1 and any comparative conditions) at 37°C, 5% CO_2_. Cells were cultured in DMEM or RPMI (Tr1 cell conditions only), both supplemented with 10% (v/v) fetal calf serum, 10 mM L-glutamine, 100 U/mL penicillin, 100 ng/mL streptomycin. Cell culture media was supplemented with Th0 (10 ng/mL IL-2), Th1 (10 ng/mL IL-2, 10 ng/mL IL-12, 10 ng/mL αIL-4), Th2 (10 ng/mL IL-2, 40 ng/mL IL-4, 10 ng/mL αIFNγ), or Th17 (20 ng/mL IL-6, 1 ng/mL TGFβ, 10 ng/mL IL-23, 10 ng/mL IL-1β, 10 ng/mL TNFα, 10 ng/mL αIL-4, 10 ng/mL αIFNγ) cytokines (eBioSciences) for CD4^+^ T cell polarization (38). After 5 days, culture supernatants were harvested and cytokine concentrations were assessed using mouse Cytometric Bead Arrays: Inflammatory Cytokine CBA Kit, Th1/Th2/Th17 Cytokine Kit, and IFNγ and IL-10 Flex Sets (BD Biosciences, Franklin Lakes, NJ, USA), according to manufacturer's guidelines.

### Generation of mixed-BM chimeric mice

Mixed bone marrow chimeric mice were generated by lethally irradiating mice with two doses of 5.5 cGy and subsequently engrafting with 10^6^ freshly isolated bone marrow cells i.v., via the lateral tail vein, as previously described(39). Irradiated recipients were engrafted with either a 50:50 or 30:70 mix of congenic (CD45.1) C57BL/6 and B6.*Bach2*^Δ*T*^ (CD45.2) bone marrow cells, as indicated in the text.

### Flow cytometry

All organ-derived mononuclear cells were prepared as previously described (28, 39). Fluorescein-conjugated mAbs against CD4 (GK1.5), CD8α (53-6.7), TCRβ (H57-597), CD11a (M1714), CD49d (R1-2), Tbet (4B10), IFNγ (XMG1.2), IL-10 (JES5-16E3), CD45.1 (A20), and CD45.2 (104) (Biolegend) were used. Dead cells were excluded from analysis using LIVE/DEAD Fixable Aqua Stain (Invitrogen), as per manufacturer's instructions. Both cell surface and intracellular staining was undertaken according to methods previously described (28), with all samples acquired on a BD LSRFortessa (BD Biosciences). Gating strategies used for analysis are outlined in Figures 3, 6. For analysis of intracellular IFNγ and IL-10, cells were stimulated for 3 h at 37°C and 5% CO_2_ in the presence of PMA (Sigma) and Ionomycin (Sigma) in addition to Brefeldin A (Sigma), as previously described (28).

### Statistical analysis

Statistical analysis was performed exclusively in GraphPad Prism 5 and 6 (GraphPad Software, La Jolla, CA). A non-parametric, un-paired Mann-Whitney test was used for comparisons between two groups. A *p*-value of < 0.05 was considered significant. Graphs depict mean ± SEM.

## Results

### T cell-specific BACH2 influences Th2 and Th17 differentiation

**Figure 1:**
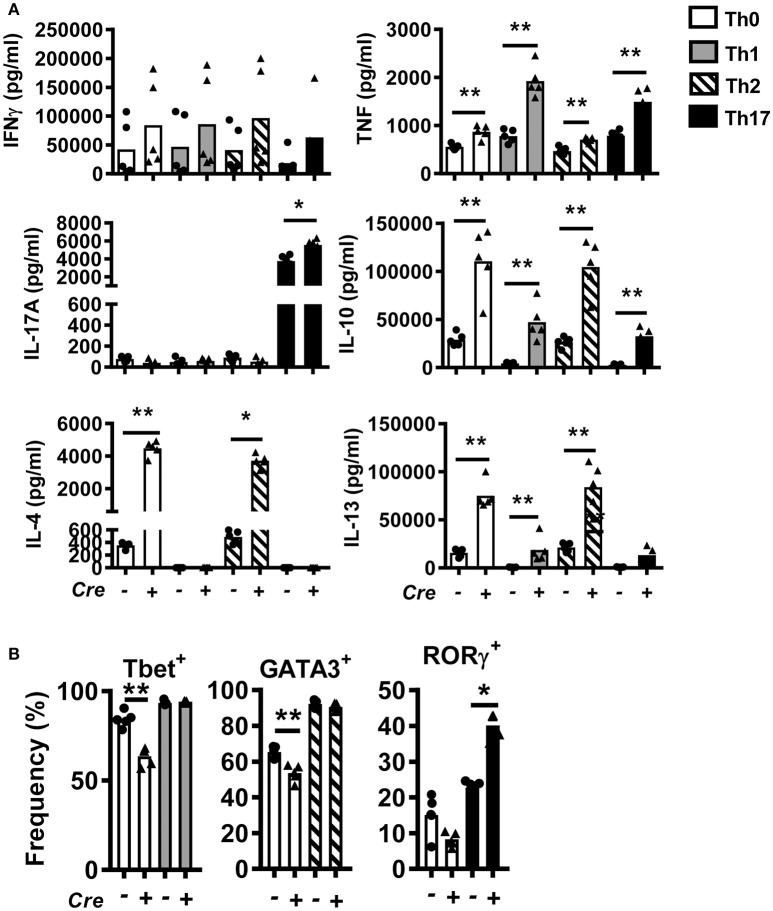
Bach2 inhibits Th17 cell development *in vitro*. CD4^+^ T cells were purified from B6.*Bach2*^Δ*T*^ (closed triangles) or B6.*Bach2*^*fl*/*fl*^ (closed dots) spleens, as indicated, and cultured with αCD28 and αCD3 mAbs for 5 days under either Th0, Th1, Th2, or Th17 cell polarizing conditions. **(A)** Cytokine levels in cell culture supernatants were measured and **(B)** frequencies of CD4^+^ T cells expressing lineage transcription factors under the various CD4^+^ T cell polarizing conditions were also assessed. *n* = 5 mice per condition in duplicate, *P < 0.05, **P < 0.01, significance assessed by Mann-Whitney U-test.

To study the role of BACH2 in CD4^+^ T cells, we generated T cell-specific *Bach2* knockout mice by crossing *Cd4*-*cre* transgenic mice with *Bach2* floxed (B6.*Bach2*^Δ*T*^) mice. Littermates lacking the *Cd4-cre* transgene (B6.*Bach2*^*fl*/*fl*^) were used as controls. CD4^+^ T cells were isolated from spleens of naïve B6.*Bach2*^*fl*/*fl*^ and B6.*Bach2*^Δ*T*^ mice and cultured with αCD3 and αCD28 mAbs in the presence of neutral (Th0), Th1, Th2, or Th17 cell polarizing cytokines. CD4^+^ T cell *Bach2*-deficiency resulted in multiple changes in cytokine production (Figure 1A), consistent with previous findings (2, 14, 15). These changes included increased TNF, IL-10, and IL-13 production under all conditions tested. In addition, B6.*Bach2*^Δ*T*^ CD4^+^ T cells produced significantly more IL-4 under Th0 and Th2 cell conditions, and significantly increased levels of IL-17A were measured under Th17 cell conditions. A notable exception to BACH2-mediated changes in CD4^+^ T cell cytokine production was IFNγ, whereby no consistent, BACH2-dependent change was observed under any cell culture condition tested. This was consistent with no change in the generation of Tbet^+^ B6.*Bach2*^Δ*T*^ CD4^+^ T cells, compared with controls, under Th1 cell conditions (Figure 1B). However, a decrease in the generation of Tbet^+^ B6.*Bach2*^Δ*T*^ CD4^+^ T cells was found under Th0 cell conditions, relative to control cells (Figure 1B). A similar observation was made for GATA3^+^ B6.*Bach2*^Δ*T*^ CD4^+^ T cells cultured under Th2 cell culture conditions, despite corresponding increased IL-4 and IL-13 production (Figure 1B). Again, a decrease in the generation of GATA3^+^ B6.*Bach2*^Δ*T*^ CD4^+^ T cells was found under Th0 cell conditions (Figure 1B). BACH2 suppressed expansion of RORγ^+^ CD4^+^ T (Th17) cells under Th17 polarizing conditions, consistent with increased levels of IL-17A in the absence of BACH2 (Figure 1B). We also observed a significant increase in IL-10 levels in all culture conditions with B6.*Bach2*^Δ*T*^ CD4^+^ T cells, compared with controls (Figure 1A). Significantly, under Th1 cell conditions, Tbet expression was maintained in B6.*Bach2*^Δ*T*^ CD4^+^ T cells (Figure 1B) and IL-10 production was increased (Figure 1A), compared with control cells, indicating BACH2 suppressed Tr1 cell development. Given that BLIMP1 is critical for CD4^+^ T cell IL-10 production (28), this finding suggests that BACH2 may inhibit CD4^+^ T cell BLIMP1-dependent IL-10 production.

### The role of BACH2 in experimental malaria

**Figure 2:**
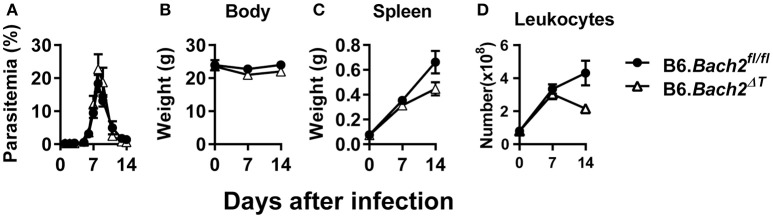
T cell-specific BACH2 does not influence disease outcome in *P. chabaudi* infection. **(A)** Blood parasitemia was measured on days 1–8 p.i. (*n* = 12 mice per group), and days 9–14 p.i. (*n* = 6 mice per group). Whole body weights **(B)**, spleen weights **(C)**, and numbers of splenic leukocytes **(D)** were measured in B6.*Bach2*^Δ*T*^ (open triangles) and B6.*Bach2*^*fl*/*fl*^ (closed circles) mice infected with *P. chabaudi* at days 0, 7, and 14 p.i., *n* = 5–7 B6.*Bach2*^Δ*T*^ and *n* = 5 B6.*Bach2*^*fl*/*fl*^ mice at each time point, mean ± SEM, significance assessed by Mann-Whitney U-test.

The outcome of *Plasmodium* infection depends on host CD4^+^ T cell responses (40–43), and BLIMP1-dependent Tr1 cell responses have a major influence on disease outcome (28). Since BACH2 influenced effector CD4^+^ T cell subset development *in vitro*, and in particular the development of Tr1 cells, we hypothesized that BACH2 would influence cellular responses during infection, and consequently, affect disease outcome. B6.*Bach2*^Δ*T*^ and control mice were infected with *Plasmodium chabaudi chabaudi* AS (*P. chabaudi*) that causes an acute, resolving infection in C57BL/6J mice (44). We chose this malaria model because there is a clear requirement for anti-parasitic Th1 cell responses to control parasite growth and Tr1 cell responses to control associated inflammation and restrict tissue pathology (45–47). We found no effect of BACH2 deficiency on control of parasite growth in this model (Figure 2A), as well as no change in body weight (Figure 2B), an indirect measure of disease severity. In addition, despite a small reduction in spleen weight (Figure 2C) and leukocyte number (Figure 2D) in B6.*Bach2*^Δ*T*^ mice, compared to control animals, this failed to reach a statistical difference at day 7 and 14 p.i.

**Figure 3:**
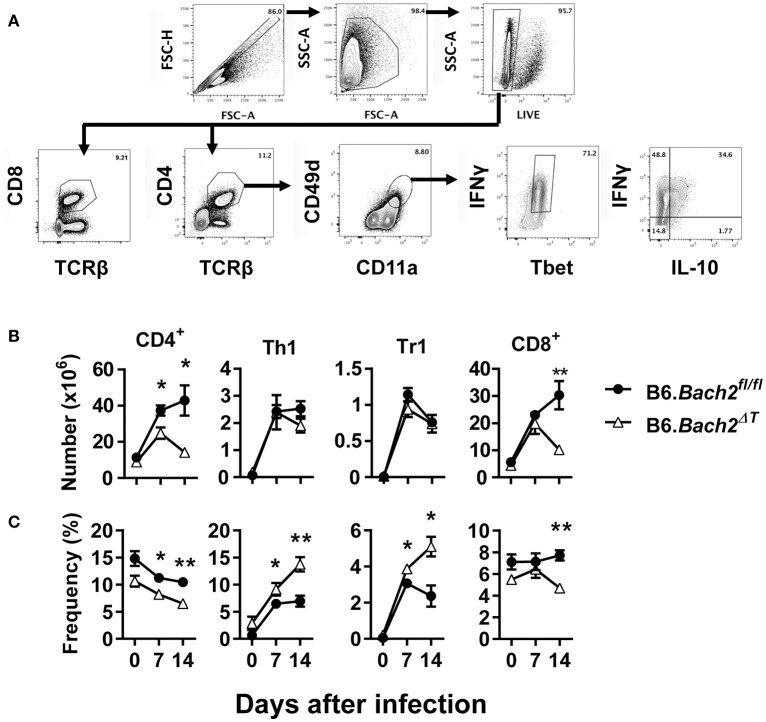
T cell-specific BACH2 supports splenic CD4^+^ T cell expansion. **(A)** Gating strategy for CD4^+^ T, CD8^+^ T, antigen experienced (CD49d^+^ CD11a^+^), Th1 (IFNγ^+^, Tbet^+^), and Tr1 (IFNγ^+^ IL10^+^) cells in the spleens of B6.*Bach2*^Δ*T*^ (open triangles) and B6.*Bach2*^*fl*/*fl*^ (closed circles) mice infected with *P. chabaudi* at 0, 7, and 14 days p.i., Numbers **(B)** and frequency **(C)** of CD4^+^ T, Th1, Tr1, and CD8^+^ T cells, as indicated, were measured by flow cytometry. *n* = 5–7 mice per time point, mean ± SEM, *P < 0.05, **P < 0.01, significance assessed by Mann-Whitney U-test.

We next investigated T cell responses to examine whether compensatory mechanisms develop in the absence of T cell BACH2 that may account for the lack of effect of BACH2-deificiency on disease outcome (Figure 3A). First, we found no statistically significant changes in the number of splenic CD4^+^ or CD8^+^ T cells in B6.*Bach2*^Δ*T*^ mice prior to infection (Figure 3B), although a small, consistent decrease in the frequency of these cell populations in the spleen was noted, compared to controls (Figure 3C). Following infection, splenic CD4^+^ T cell numbers expanded in B6.*Bach2*^Δ*T*^ and control mice over the first 7 days of infection, but then declined over the following 7 days in B6.*Bach2*^Δ*T*^ mice, while continuing to increase in control animals (Figure 3B). Although no change in the number of B6.*Bach2*^Δ*T*^ Th1 and Tr1 cells was found, compared to controls, there was an increased frequency of both these cell populations over the course of infection, relative to B6.*Bach2*^*fl*/*fl*^ cells. Given that CD8^+^ T cells from B6.*Bach2*^Δ*T*^ also lack Bach2 expression, we measured these cells and found a similar pattern of expansion and contraction as seen with corresponding CD4^+^ T cells (Figures 3B,C). Thus, despite significant changes in numbers and frequencies of B6.*Bach2*^Δ*T*^ T cell subsets following *P. chabaudi* infection, including increased frequencies of Th1 and Tr1 cells, there was little impact on disease outcome, suggesting that compensatory immune mechanisms were activated *in vivo*. Alternatively, it is possible that T cell-specific BACH2 is dispensable for control of parasite growth and disease outcome.

### A cell intrinsic role for BACH2 in experimental malaria

**Figure 4:**
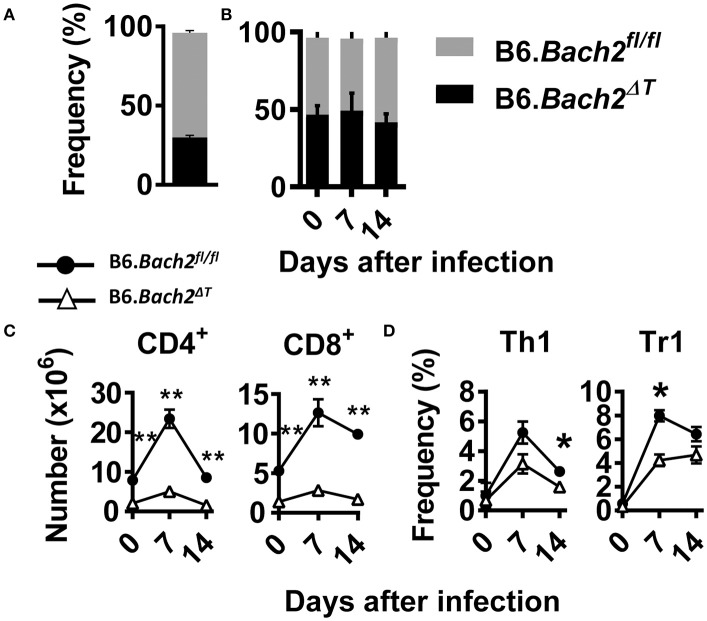
Cell intrinsic BACH2 supports T cell development and expansion. B6.*Bach2*^Δ*T*^ (CD45.2):B6.*Bach2*^*fl*/*fl*^ (CD45.1) (50:50) mixed bone marrow chimeric mice were generated in lethally irradiated B6.*Rag1*^−/−^ mice and engraftment measured in splenic leukocytes 12 weeks later **(A)**. Subsequently, these mice were generated by injecting a 70:30 mix of bone marrow from *B6.Bach2*^Δ*T*^ and B6.*Bach2*^*fl*/*fl*^ mice into irradiated B6.*Rag1*^−/−^ mice, and the proportions of CD45.2 and CD45.1 leukocytes in the spleen were measured before *P. chabaudi* infection and 7 and 14 days p.i., as indicated **(B)**. Numbers of CD4^+^ T and CD8^+^ T cells **(C)**, as well as frequency of Th1 and Tr1 cells **(D)** in the spleen at days 0, 7, and 14 p.i., as indicated, were measured by flow cytometry. *n* = 6 mice per group per time point. Mean ± SEM, *P < 0.05, **P < 0.01, significance assessed by Mann-Whitney U-test.

The above findings identify several important roles for BACH2 in T cell subset development and/or survival during experimental malaria. To test whether cell intrinsic roles for BACH2 in different T cell subsets were responsible, we generated B6.*Bach2*^Δ*T*^ (CD45.2):B6.*Bach2*^*fl*/*fl*^ (CD45.1) (50:50) mixed bone marrow chimeric mice by injecting bone marrow from B6.*Bach2*^Δ*T*^ and B6.*Bach2*^*fl*/*fl*^ mice into lethally irradiated B6.*Rag1*^−^^/–^ mice (Figure 4). To generate animals with an approximate 50:50 mix of leukocytes, we had to increase the ratio of B6.*Bach2*^Δ*T*^ :B6.*Bach2*^*fl*/*fl*^ bone marrow to 70:30 because the former cells did not engraft as well as the control cells when injected in an equal ratio (Figure 4A). This result indicated a fundamental role for T cell BACH2 in the efficient reconstitution of immune systems following lethal irradiation. After 12 weeks of engraftment, similar B6.*Bach2*^Δ*T*^ and B6.*Bach2*^*fl*/*fl*^ leukocyte reconstitution was measured (Figure 4B). However, despite the increased ratio of B6.*Bach2*^Δ*T*^ bone marrow in grafts resulting in relatively even leukocyte reconstitution from B6.*Bach2*^Δ*T*^ and B6.*Bach2*^*fl*/*fl*^ bone marrow sources, we still found a reduction in B6.*Bach2*^Δ*T*^ CD4^+^ T cells and CD8^+^ T cell numbers in the spleens of uninfected mice, compared to controls (Figure 4C). This reduction was maintained following infection with *P. chabaudi* (Figure 4C). We next measured Th1 and Tr1 cell frequencies in mice over the course of *P. chabaudi* infection and found small reductions in *Bach2*-deficient, splenic Th1 and Tr1 cells at days 14 and 7 p.i., respectively (Figure 4D). Together, these results show that *Bach2* expression by T cells plays an important role in hematopoietic cell development, and in particular, the generation of CD4^+^ and CD8^+^ T cells. Furthermore, T cell intrinsic BACH2 is needed for efficient expansion of Th1 and Tr1 cells during experimental malaria. This latter result was surprising given the increased expansion of Th1 and Tr1 cells in *P. chabaudi*-infected B6.*Bach2*^Δ*T*^ mice, compared to control mice, and indicates both cell extrinsic and intrinsic roles for BACH2 in Tr1 cell expansion during infection.

### The role of BACH2 in experimental visceral leishmaniasis

**Figure 5:**
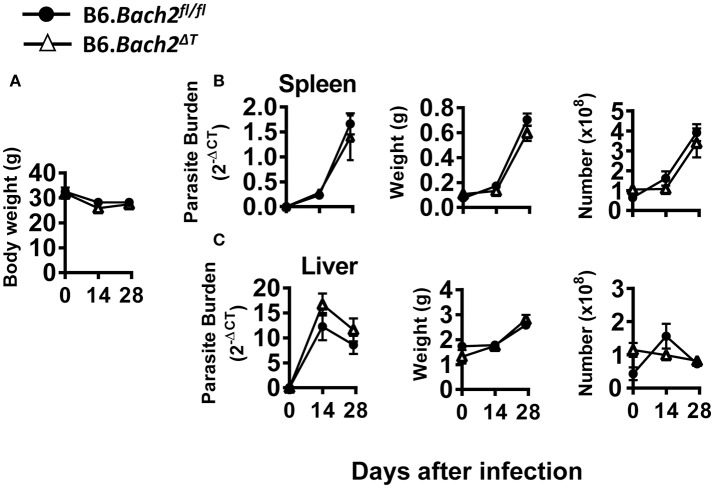
T cell-specific BACH2 deficiency does not influence disease outcome in *L. donovani* infection. B6.*Bach2*^Δ*T*^ (open triangles) and B6.*Bach2*^*fl*/*fl*^ mice (closed circles) mice were infected with *L. donovani* and assessed for body weight **(A)**, as well as spleen **(B)** and liver **(C)** parasite burden, organ weight and leukocyte number, as indicated, at days 0, 14, and 28 p.i., *n* = 3–7 mice per group per time point, mean ± SEM.

The above results in experimental malaria were unexpected given the role of BACH2 in CD4^+^ T cell subset development identified in *in vitro* experiments (Figure 1). Therefore, we also investigated the role of BACH2 in visceral leishmaniasis (VL) caused by infection with the human protozoan parasite *Leishmania donovani* to establish how broadly applicable our findings were. This C57BL/6J mouse model of VL is characterized by an acute, resolving infection in the liver, accompanied by the development of a chronic infection in the spleen (48, 49). Thus, in addition to examining immune responses in the spleen, we were also able to study these responses in the liver—a non-haematopoietic organ in adult mice. B6.*Bach2*^Δ*T*^ mice infected with *L. donovani* had little difference in spleen, liver or body weight, parasite burdens or leukocyte numbers over the first 28 days of infection, compared to control mice (Figure 5). Interestingly, although hepatic leukocyte numbers expanded in control B6.*Bach2*^*fl*/*fl*^ mice over the first 14 days of infection, this was not observed in B6.*Bach2*^Δ*T*^ mice, which appeared to have higher leukocyte numbers in the liver prior to infection (Figure 5C).

**Figure 6:**
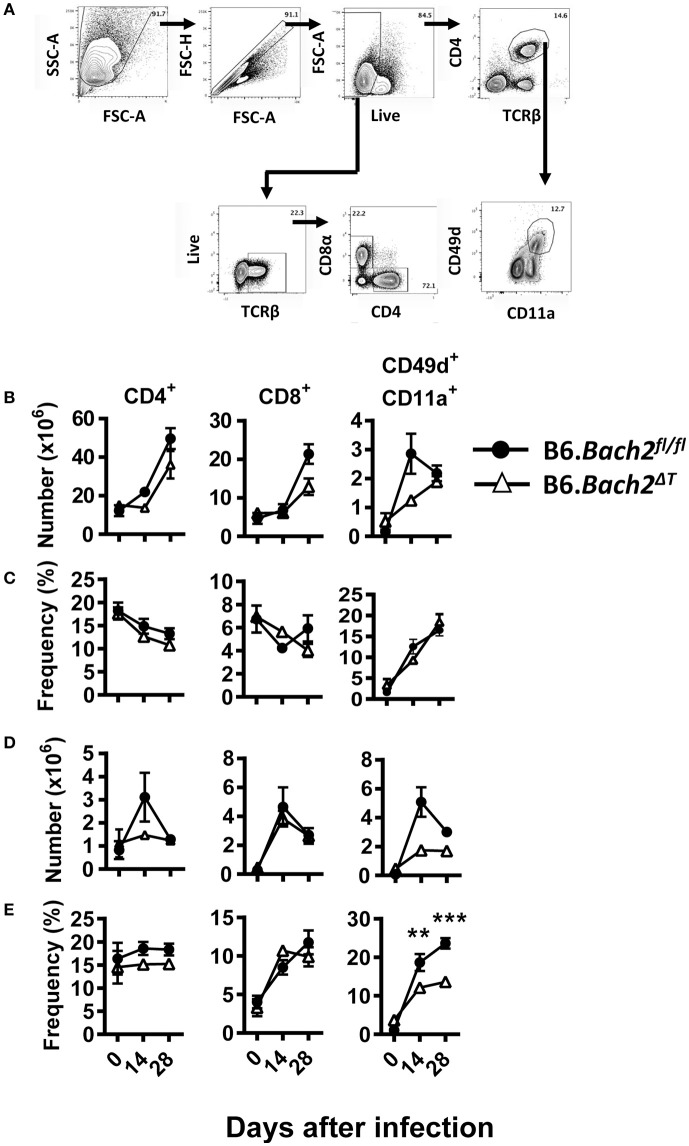
T cell-specific BACH2 influences tissue-specific expansion of antigen experienced CD4^+^ T cells following *L. donovani* infection. **(A)** Gating strategy for CD4^+^ T, CD8^+^ T, and antigen-experienced (CD49d^+^ CD11a^+^) CD4^+^ T cells in the spleen **(B,C)** and liver **(D,E)** of B6.*Bach2*^Δ*T*^ (open triangles) and B6.*Bach2*^*fl*/*fl*^ (closed circles) mice infected with *L. donovan* at 0, 14, and 28 days p.i., The numbers **(B,D)** and frequencies **(C,E)** of CD4^+^ T, CD8^+^ T, and antigen-experienced CD4^+^ T cells, as indicated, were measured by flow cytometry. *n* = 4–7 mice per time point, mean ± SEM, **P < 0.01, ***P < 0.001 significance assessed by Mann-Whitney U-test.

Despite no significant changes in spleen and liver CD4^+^ and CD8^+^ T cell number or frequency (Figures 6A–D), we did find tissue-specific changes in recently antigen-experienced (CD49d^+^ CD11a^+^) CD4^+^ T cells in B6.*Bach2*^Δ*T*^ mice. There was a significant reduction in the frequency of recently antigen-experienced CD4^+^ T cells at days 14 and 28 p.i., in the liver (Figure 6E), but not the spleen (Figure 6C), compared to B6.*Bach2*^*fl*/*fl*^ mice. This finding suggests a role for BACH2 in the expansion and/or survival of activated CD4^+^ T cells entering the liver during infection. However, despite these tissue-specific changes in recently antigen-experienced CD4^+^ T cells, there was little impact of BACH2-deficiency on disease outcome in this second pre-clinical model of protozoan parasitic infection.

## Discussion

Our studies on BACH2 using T cell-specific knockout mice have demonstrated a T cell intrinsic role for BACH2 in T cell expansion and/or survival during *P. chabaudi* and *L. donovani* infection. These findings are consistent with those reported in other studies (2, 20, 50), and add to our understanding about the role of BACH2 in various CD4^+^ T cell subsets. Our results indicate that T cell specific BACH2 deficiency most affects Th17 and Th2 cell development and maintenance. However, malaria and VL are not strongly influenced by these CD4^+^ T cell subsets, which may explain why a relatively minor effect on disease outcome was found in pre-clinical models of these diseases. Thus, future studies on T cell BACH2 might best be directed toward disease where these CD4^+^ T cell subsets are more important, such as multiple sclerosis or asthma, which are Th17 and Th2 cell-dependent, respectively. However, studies with mixed bone marrow chimeric mice also indicated both cell extrinsic and intrinsic roles for BACH2 in Th1 and Tr1 cell development, indicating that compensatory mechanisms may emerge in the absence of BACH2 during parasitic infection to initiate and maintain anti-parasitic immunity.

Our recent assessment of CD4^+^ T cells from human peripheral blood showed that *BACH2* was down regulated during *P. falciparum* infection (51). This was consistent with results from other studies that indicate that *BACH2* needs to be down regulated to allow T cells to be activated and function effectively (2, 37, 50). Hence, another reason we may not have seen major changes in infected mice with *Bach2*-deficient T cells was because gene expression was already down regulated. Any differences would likely occur during the initial response to infection, and therefore, studying earlier time points post-infection might reveal these effects. Also, given the down-regulation of *Bach2*, it may be informative to examine the impact of *Bach2* over-expression during infection in future studies.

We found that T cell-specific *Bach2* expression suppressed Th2 and Th17 cell development and/or activation. Similar results were observed in experiments involving cells from mice with ubiquitous *Bach2*-deficiency (2, 15). However, a problem with interpreting results from these experiments was the difficulty of excluding the possibility that *Bach2*-deficiency in a non-T cell population impacted T cell development, and was therefore responsible for changes observed. Our data indicate that T cell intrinsic BACH2 plays an important role in regulating Th2 cell cytokine production. However, other studies have shown that BACH2 also suppressed Th1 and Th17 cytokine production (2, 37). Our data supports this role in Th17 cells, but not in Th1 cell cytokine production.

Previous work has shown that BACH2 regulates BLIMP1 activity (52). Indeed, we recently showed that BLIMP1 was required for Tr1 cell development in experimental malaria and VL (28), and results from the current study showed increased frequencies of splenic Th1 and Tr1 cells in *P. chabaudi*-infected B6.*Bach2*^Δ*T*^ mice, despite reductions in overall CD4^+^ T cells numbers, relative to littermate controls. However, as mentioned above, these increased Th1 and Tr1 B6.*Bach2*^Δ*T*^ cell frequencies were not observed in mixed bone marrow chimeric mice. Given that this latter setting is one where haematopoietic cells from B6.*Bach2*^Δ*T*^ and B6.*Bach2*^*fl*/*fl*^ donors compete to fill various tissue niches and expand following infection, our results indicate an important role for BACH2 in T cell development, tissue recruitment and/or retention, independent of T cell activation, as well as a distinct role in CD4^+^ T cell differentiation following infection. The balance between these different roles is likely to determine disease outcome in different settings, depending on the specific requirements of CD4^+^ T cell subsets needed for protection. Therefore, although BACH2 may interact with BLIMP1 to influence CD4^+^ T cell development and differentiation (52), our results indicate that this interaction plays a limited role in determining the outcome of infection with *P. chabaudi* or *L. donovani*.

As mentioned earlier, BACH2 impacts the terminal differentiation of CD8^+^ T cells by controlling availability of transcription factor binding sites, and in particular, by controlling AP-1 availability (50). It is possible that BACH2 acts in a similar manner to influence development of different CD4^+^ T cell populations. NF-kB and AP-1 family members are required for full Th2 cell differentiation, and the Th2 cell cytokines IL-4 and IL-13 have AP-1 binding sites in their promoters (53, 54). Thus, BACH2 may inhibit the expression of these genes by preventing AP-1 binding, and therefore prevent their transcription and subsequent expression. Support for this mechanism came from studies using an AP-1 decoy molecule, which was used to block IL-4 and IL-13 production, and ameliorate disease symptoms in a model of asthma (55). NF-kB/AP-1 binding was also increased in rheumatoid arthritis patients (56), and similarly, *NF-kB/AP-1* expression was correlated with type-1 diabetes pathogenesis (57). Given that both diseases are associated with *BACH2* dysregulation (8, 58), this may contribute to aberrant *NF-kB/AP-1* expression. Others also hypothesize that the homology between BACH2 and AP-1 sequences may allow BACH2 to bind in place of AP-1 in umbilical cord blood, where *BACH2* was shown to regulate IL-2 expression (59). Therefore, one mechanism by which BACH2 may influence T cell differentiation and disease outcome is by competing with AP-1 for DNA binding.

The absence of T cell BACH2 may also promote changes in the regulation of apoptosis, as the JNK/AP-1 pathway has been associated with apoptosis in synovial cells in rheumatoid arthritis (60), and several studies have implicated BACH2 in promoting apoptosis. For example, BACH2 facilitated apoptosis in B cells by suppressing anti-oxidative and anti-apoptotic genes (61, 62). However, the loss of BACH2 caused enhanced CD8^+^ T cell apoptosis 5–10 days after viral infection (50), indicating a different role in T cells. This may help explain the loss of *Bach2*-deficient CD4^+^ T cells we observed in the spleen during *P. chabaudi* infection, although this remains to be tested. Furthermore, apoptosis was found to be associated with a reduction in anti-apoptotic Bcl-2 family proteins Bcl-xL and Mcl-1 (50). Interestingly, BACH2 has been associated with Bcl-2 and Mcl-1 in other disease settings (58). Therefore, although BACH2 is pro-apoptotic in B cells, it appears to act as an anti-apoptotic molecule in CD8^+^ T cells, and if it has a similar role in CD4^+^ T cells, this may help to explain some of our findings.

In summary, we showed BACH2 is an important intrinsic factor in CD4^+^ T cells during differentiation *in vitro* and during parasitic infection. We showed that T cell-specific BACH2 modulates Th2 and Th17 cell differentiation, and suppressed effector CD4^+^ T cell responses during infection. However, BACH2 was especially important for the expansion and/or maintenance of splenic CD4^+^ T cells during *P. chabaudi* infection, and a possible mechanism for this role may be via regulation of AP-1 binding to cell lineage-specific genes and anti-apoptotic genes. Our results indicate that BACH2 either has a minor role in disease outcome during malaria and VL or compensatory mechanisms for BACH2 function are effectively activated following infection with the protozoan parasites that cause these diseases.

## Ethics statement

All animal procedures were conducted with the approval of the QIMR Animal Ethics Committee under the animal ethics number A02-634M and in accordance with the Australian Code of Practice for the Care and Use of Animals for Scientific Purposes (Australian NHMRC, Canberra).

## Author contributions

CLE and CRE designed, performed and analyzed the work, and wrote the paper. MdO, FdLR, RK, SN, YW, and FA performed the work and analyzed the data. KK, TK, TS, and AK provided reagents and expert advice on experimental design and interpretation of data.

### Conflict of interest statement

The authors declare that the research was conducted in the absence of any commercial or financial relationships that could be construed as a potential conflict of interest.
